# Assessment of cellular and molecular metrics for dose selection in an *in vivo* comet assay: A case study with MDI

**DOI:** 10.1002/em.22457

**Published:** 2021-08-26

**Authors:** Zhiying Ji, Matthew W. Koehler, Andrew B. Scott, Matthew J. LeBaron

**Affiliations:** ^1^ The Dow Chemical Company, Toxicology & Environmental Research & Consulting Midland Michigan USA

**Keywords:** apoptosis, cytotoxicity, *in vivo* comet, MDI

## Abstract

The *in vivo* comet assay can evaluate the genotoxic potential of a chemical in theoretically any tissue that can be processed to a single cell suspension. This flexibility enables evaluation of point‐of‐contact tissues using a relevant route of test material administration; however, assessing cytotoxicity is essential for the interpretation of comet results. Histopathological evaluation is routinely utilized to assess cytotoxicity, but temporal‐ and cell‐specific considerations may compromise applicability to the comet assay. In the present study, 1,1′‐methylenebis(4‐isocyanatobenzene) (4,4'‐MDI) was administered to rats for 6 h by nose‐only inhalation, and the comet assay was conducted to evaluate genotoxicity in the site‐of‐contact tissue (bronchoalveolar lavage cells) and distal tissues (liver and glandular stomach). Given the reactive nature of MDI, cellular and molecular metrics at the site‐of‐contact‐ including inflammation, macrophage activation, apoptosis/necrosis, and oxidative stress‐ were used to set appropriate exposure concentrations, in addition to the standard systemic measures of toxicity. In the range‐finding study, a concentration of 4 mg/m^3^ was considered the maximum noninflammatory concentration; hence target concentrations of 2, 5, and 11 mg/m^3^ were selected for the comet study. In the lung lavage, MDI exposure substantially increased total protein and β‐glucuronidase, along with cellular apoptosis. Although MDI did not increase the comet assay response (% tail DNA) in any of the tissues examined, the positive control (ethyl methanesulfonate, EMS) significantly increased % tail DNA in all tissues. In total, these data indicate that appropriate cellular and molecular measurements may facilitate dose selection to discern cellular status in the comet assay.

## 1 INTRODUCTION

The potential consequences of a chemical's interaction with genetic material generally falls into two categories—mutagenic and genotoxic. Specifically, mutagenicity is defined as heritable alterations in DNA (i.e., specific base pair sequence and/or a structural or numerical chromosome alteration), whereas the broader term genotoxicity includes genetic alterations that may or may not be transmissible from cell‐to‐cell or generation‐to‐generation. The *in vivo* comet assay is an indicator test for genotoxicity that assesses only primary DNA damage and not necessarily the potential heritable consequences of that damage. This primary damage can, in theory, be repaired, result in cell‐stasis or cell‐death, or proceed to a bona fide mutation. The most commonly utilized comet assay is the *in vivo* alkaline single‐cell gel electrophoresis assay (OECD_TG489, 2016) and, in this format, can detect single and double DNA strand breaks as well as alkali‐labile sites in the genome. An obvious benefit of the *in vivo* comet assay is the ability to evaluate essentially any tissue (or theoretically cell type), including specific point‐of‐contact sites depending on the route of administration of a test material.

In addition to the *in vivo* comet assay being an indicator assay for potential genotoxicity, it is well established that an increase in a comet response may be the result of coincident (or exclusive) cytotoxicity (Burlinson et al., 2007; OECD, 2014). This apoptosis/necrosis signature has been correlated with increases in a comet tail and suggested not to be readily discerned from a true genotoxicity signature (Guerard et al., 2014; Lorenzo et al., 2013; OECD, 2014). Given this, and as indicated by the OECD_TG489 (2016), a thorough assessment of cytotoxicity should be undertaken during the conduct of an *in vivo* comet assay. While histopathological assessment of the specific tissue examined in the comet assay is considered the gold standard (Burlinson et al., 2007; OECD_TG489, 2016; Uno et al., 2015), it is clear that the temporal nature of cytotoxicity, as determined by different metrics, may be appreciably altered with variable timing (Cobb et al., 1996; Jones et al., 1997; Majno & Joris, 1995; Vasquez, 2012). Furthermore, not all cell types utilized in the comet assay are amenable to histopathology for cellular and tissue‐contextual evaluation, for example, when utilizing nucleated peripheral blood or lung lavage cells. In these cases, and as indicated in the OECD test guideline, other measures of cellular status such as apoptosis, necrosis, and general metabolic competency may be considered.

**FIGURE 1:**
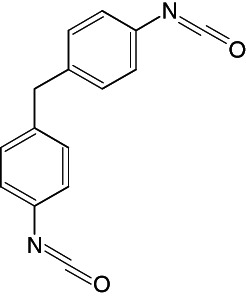
Structure of 1,1′‐methylenebis(4‐isocyanatobenzene) (MDI)

The aromatic isocyanate 1,1′‐methylenebis(4‐isocyanatobenzene) (aka 4,4′ diphenylmethane diisocyanate, hereafter referred to as MDI; Figure 1) is a highly utilized industrial precursor used for production of polyurethane foams and adhesives. The chemical nature, toxicity data, and structure–activity relationship indicates a reactive compound that must be produced, distributed, and utilized with care. In animal studies, acute exposure to respirable MDI resulted in pulmonary inflammation and chronic exposure at 6 mg/m^3^ (but not lower) increased the incidence of lung tumors in rats (Reuzel et al., 1990; Reuzel et al., 1994). MDI was negative for mutagenicity in *Salmonella typhimurium* strains TA98, TA100, TA1535, and TA1537 in the presence or absence of metabolic activation (Zeiger et al., 1987). Multiple *in vivo* micronucleus assays with MDI were negative for mutagenicity (Pauluhn et al., 2001), including a study in mice where MDI‐hemoglobin adducts were identified in the blood, demonstrating systemic exposure to the inhaled MDI (Lindberg et al., 2011). Taken together, the weight of evidence supports non‐genotoxic pathway for carcinogenicity observed in rats (Pauluhn et al., 2001), although a point‐of‐contact evaluation of the genotoxic potential of MDI had not been undertaken.

The present study was conducted to further assess the acute response to MDI, including at the point‐of‐contact when administered via inhalation. The intent was, ideally, to establish and utilize cellular and molecular data to supplement standard parameters to aid in dose−/concentration‐level selection along with evaluation of the genotoxic hazard potential of MDI in a Good Laboratory Practices (GLP) and OECD Test Guideline‐compliant *in vivo* comet assay.

## 2 MATERIALS AND METHODS

### 2.1 Dose‐range finding study and evaluation of cellular and molecular parameters

#### 2.1.1 Animals and chemicals

As an initial phase, a study was conducted to define a maximum tolerated concentration (MTC) of inhaled MDI for a subsequent guideline comet study. Male Crl:WI(Han)/Wistar rats were purchased from Charles River (Raleigh, NC) and were 11 weeks old at the time of exposure. MDI (CAS # 101‐68‐8) was purchased from Covestro Deutschland AG, Ward Hill, MA (Lot No. P4DB005186, purity 98.89%). This phase of the study was conducted in the Toxicology & Environmental Research & Consulting Laboratory of The Dow Chemical Company, Midland, MI, after approval by the Instutitional Animal Care and Use Committee (IACUC).

#### 2.1.2 Experimental exposures

Before administration of test material, animals were stratified by body weight and then randomly assigned to four treatment groups (12/group) using a computer program designed to increase the probability of uniform group mean weights and standard deviations at the start of the study. Rats were exposed to MDI for 6 h using a nose‐only inhalation exposure system to target concentrations of 0, 5, 10, or 20 mg/m^3^; actual concentrations were 0, 4, 12, or 27 mg/m^3^. The concentration of aerosol present in each chamber was determined gravimetrically at least once during the 6‐h exposure period. The average or time‐weighted average (TWA) exposure concentrations were calculated from the gravimetric measurements. The aerodynamic particle size distribution was determined once during the exposure period.

A dry aerosol of test material was generated using a BLAM (Blaustein Atomizing Module, CH Technologies, Westwood, NJ) aerosol generator containing molten MDI. The BLAM was heated using heat tape and supplied with a preheated stream of filtered compressed air. The temperature and the volumetric flow of air through the BLAM were adjusted as needed. The output of the BLAM was directed to a central mixing (elutriation) chamber, which was connected to each exposure chamber. Target exposure concentrations were achieved by dilution of the test aerosol from the mixing chamber.

#### 2.1.3 Bronchoalveolar lavage

Immediately following exposure or 18 h postexposure, animals were anesthetized with a mixture of isoflurane vapors and medical grade oxygen and euthanized by exsanguination by severing the abdominal aorta and/or associated vessels. The thoracic cavity was opened, the trachea cannulated, and the entire lung with trachea and extrapulmonary airways attached was removed. Bronchoalveolar lavage (BAL) was performed on all animals. The entire lung from each animal was lavaged in two stages. The first lavage was performed with two volumes (25 μl/gram body weight) of calcium‐ and magnesium‐free Hanks balanced salt solution (HBSS); these samples were pooled. A second round of lavage was performed with four volumes (25 μl/gram body weight) of calcium‐ and magnesium‐free HBSS, and these four lavage volumes from each animal were pooled. The lavage samples were placed on wet ice until processed for analysis.

#### 2.1.4 Cellular and molecular metrics

The first lavage sample from each animal was centrifuged at approximately 300*g* for 5 min to pellet the BAL cells (BALC). The cell‐free supernatant lavage fluid (BALF) from the first (pooled) lavage samples was removed and aliquots of the fluid were used for analysis of total protein, lactate dehydrogenase (LDH), alkaline phosphatase (ALP), β‐glucuronidase, and reduced/oxidized glutathione (GSH/GSSG). Aliquots for GSH/GSSG analysis were stabilized in 10% trichloroacetic acid in water.

Total protein, LDH, and ALP were analyzed using a cobas c311 Clinical Chemistry Analyzer (Roche Diagnostics, Indianapolis, IN). β‐Glucuronidase was analyzed using a commercially available ELISA kit (LifeSpan BioSciences, Seattle, WA) according to manufacturer's recommendations without any modifications. For the quantitation of GSH and GSSG, the processed samples were thawed and diluted as needed for analysis using high performance liquid chromatography (HPLC) with tandem mass spectrometry (MS/MS) detection according to the method described by Zhang et al. (2015).

The cell pellets from both lavages (BAL cells) were resuspended in Dulbecco's modified Eagle's medium (DMEM)/high modified and combined and analyzed to determine the cellular endpoints, including total number and types of cells, apoptosis (via Annexin‐V and Caspase‐3, see below), and gene expression by RT‐PCR. The lavage fluid from the second lavage was discarded.

A manual total white blood cell count was performed on an aliquot of the resuspended cell pellets using a hemocytometer. A concentration of approximately 5 × 10^4^–5 × 10^5^ cells/ml was used to prepare cell smears using a Cytospin 3 cytocentrifuge (Shandon, Pittsburgh, PA). Aliquots of the cell suspension were placed in cytospin funnels and centrifuged at approximately 800 rpm for 5 min to deposit the cells on the slide (i.e., cell smear). The cell smears were air dried overnight and stained with Modified Wright‐Giemsa Stain Pack (Richard‐Allan Scientific, Kalamazoo, MI). The percent composition of each cell type (macrophage, neutrophil, eosinophil, and lymphocyte) was determined and the number of each cell type/ml lavage fluid was determined by multiplying the percent occurrence of each cell type by the total cells/ml determined previously.

Total RNA was extracted from an aliquot of the BALC using the Qiagen RNeasy kit (Qiagen, Germantown, MD) following the manufacturer's protocol. RNA quantity and quality were assessed by a NanoDrop ND‐1000 Spectrophotomer (Thermo Fisher Scientific Waltham, MA), and only samples with an OD 260/280 ratio greater than 1.8 were used. Targeted gene expression analysis was conducted using an Applied Biosystems ViiA 7 real time PCR system using Applied Biosystems TaqMan gene expression (Thermo Fisher Scientific Waltham, MA). The following genes were selected to aid in understanding the potential altered cellular pathways and were analyzed in a singleplex reaction: Cytokine Markers (*Tnf‐a*: Rat ABI Taqman ID: Rn01424675_m1, *Il‐1a*: Rat ABI Taqman ID: Rn00566700_m1, *Il‐6*: Rat ABI Taqman ID: Rn01410330_m1, *Il‐10*: Rat ABI Taqman ID: Rn99999012_m1, *Ifn‐γ*: Rat ABI Taqman ID: Rn00594078_m1, *Mip‐2*: Rat ABI Taqman ID: Rn00586403_m1, *Mcp‐1*: Rat ABI Taqman ID: Rn00580555_m1), Apoptosis Markers (*Bcl‐2*: Rat ABI Taqman ID: Rn99999125_m1, *Bax*: Rat ABI Taqman ID:Rn01480161_g1), Oxidative Stress Markers (*Aox1*: Rat ABI Taqman ID: Rn00571242_m1, *Gpx1*: Rat ABI Taqman ID: Rn00577994_g1, *Nrf2a*: Rat ABI Taqman ID: Rn01767215_m1), and the housekeeping gene *β‐actin* (*Actb*: Rat ABI Taqman ID: Rn00667869_m1). Gene expression was quantified using the comparative threshold cycle (Ct) method (ΔΔCt) (Schmittgen & Livak, 2008). The data were reported as fold‐change when compared to the control group. Statistical analysis was not performed for gene expression data.

FITC Annexin V Apoptosis Detection Kit I (BD Biosciences, cat# 556547) was used for the Annexin V analysis. Manufacturer's instructions were followed with minor modifications. In brief, approximately 0.2 × 10^6^ cells were transferred into 5 ml cold 1× Dulbecco's phosphate‐buffered saline (1× DPBS) (with Ca^2+^) and pelleted by centrifugation at 500*g* for 10 min. Supernatant was discarded via aspiration, leaving approximately 100 μl. Cells were resuspended with 2 ml cold 1× binding buffer and pelleted by centrifugation at 500*g* for 10 min. Supernatant was discarded via aspiration, leaving approximately 200 μl. Ten microliters of FITC Annexin V and 10 μl of propidium iodide (PI) were added, vortexed, and cells were incubated at room temperature in the dark for 15 min. After incubation, 300 μl of 1× binding buffer was added into each sample. Samples were transferred into flow tubes and kept on ice (protected from light). Samples were analyzed using a Gallios flow cytometer (Beckman Coulter, Brea, California) with Gallios Cytometer 1.2 software. Cells were captured based upon forward scatter and side scatter. The Annexin V negative/positive was defined upon the FITC fluorescent reading (excitation/emission = 488/509 nm, PMT FL1, blue laser). The PI negative/positive was defined upon PMT FL3 (excitation/emission = 488/620 nm, blue laser). The flow cytometry protocol (voltage, amplification, compensation, and quadrants [negative/positive cut‐offs], etc.) was set up using unstained cells, cells only stained with FITC Annexin V, or cells only stained with PI.

FITC Active Caspase‐3 Apoptosis Kit (BD Biosciences, cat# 550480) was used for the Caspase‐3 analysis. Manufacturer's instructions were followed with minor modifications.

#### 2.1.5 Statistical analyses

Analyses were conducted in SAS/STAT software, version 9.2 of the SAS System, Copyright 2002‐2008, SAS Institute. BAL fluid (BALF) parameters (APL, LDH, total protein, β‐glucuronidase, GSH, GSSG, total GSH, total GSSG, total oxidative stress, total GSH/GSSG, and total GSH equivalents) and BALC parameters (total cells, percent macrophages, and percent neutrophils) were evaluated using analysis of variance (ANOVA) to compare each endpoint between exposure groups within each time point. If the concentration effect was significant at *p* ≤ .05, a Dunnett's test was used to compare the test concentrations to the control group at *p* ≤ .05.

### 2.2 Main study with application of cellular and molecular metrics and comet assay

#### 2.2.1 Animals and chemicals

Male Crl:WI (Han)/Wistar rats were purchased from Charles River Laboratories, Raleigh, NC and were approximately 7 weeks old at the initiation of the dose administration. MDI was purchased from Covestro LLC, Pittsburgh, PA (Lot No. P4DB005186, purity 98.89%). The positive control substance ethyl methanesulfonate (EMS) was purchased from Sigma‐Aldrich (Batch No. BCBQ0451V). This phase of the work was conducted at Charles River Laboratories, Ashland, OH after approval by the IACUC, and the comet portion of the study undertaken by BioReliance, Rockville, MD.

#### 2.2.2 Experimental exposures

The animals judged suitable for assignment to the study were selected for use in a computerized randomization procedure based on body weight stratification in a block design. Groups 1–4 each consisted of 12 males and Group 5 consisted of six males.

Rats were exposed to MDI at targeted concentrations of 0, 2, 5, or 11 mg/m^3^ via a single, 6h nose‐only inhalation exposure; actual concentrations were 0, 2.5, 4.9, or 12 mg/m^3^. A concurrent control group received filtered air on a comparable regimen. An additional group (Group 5) of six animals was administered EMS at 200 mg/kg via oral gavage as the comet assay positive control.

Nose‐only inhalation exposures were conducted under dynamic airflow conditions using two tier 7.9 L stainless steel flow‐pass nose‐only exposure chambers. Four dedicated exposure systems were used: one for the filtered‐air control group and one each for the MDI exposure groups. An aerosol was generated using single‐jet collison nebulizers filled with the test material and heated to approximately 80°C to melt and maintain the MDI test material in liquid form. The resulting aerosol from each nebulizer was delivered to a mixing plenum where it was mixed with additional dry dilution air. In order to permit reduction of the aerosol generated to achieve the target MDI dry aerosol concentrations a portion of the aerosol output from each nebulizer and/or mixing plenum was directed to the exposure system exhaust. The remaining aerosol within each mixing plenum was delivered to a “T”‐fitting located prior to the chamber inlet where it was mixed with additional dry air prior to entering each nose‐only exposure to achieve the target chamber aerosol exposure concentration. Chamber oxygen content was 20.9%. All nose‐only chamber exhaust passed through a Solberg canister filter prior to entering the facility exhaust system.

Nominal exposure concentrations were not calculated for this study due to the nature of each aerosol generation system, where a large portion of the test substance aerosol was removed prior to the final dilution to the target concentration; however, the amount of test substance used during the exposure was calculated by weighing each test substance nebulizer prior to and after exposure. Aerosol exposure concentrations were measured using standard gravimetric methods. Sample flow was measured using a mini‐Buck calibrator. The mass concentration (in mg/m^3^) was calculated from the filter weight difference divided by the sample volume. Samples were collected at least 4–6 times during each exposure for the low, mid, and high exposure chambers and once for the control chamber system. Each test substance exposure atmosphere was continuously monitored for aerosol concentration using a light scattering type real time aerosol monitor. The real‐time aerosol monitors were not intended to define exposure concentration but were used to provide exposure personnel with an indication of approximate aerosol concentration for guidance in making appropriate system adjustments and achieving the most stable exposure concentration. Aerosol particle size measurements were conducted using a 7‐stage brass cascade impactor. Aerosol particle size measurements were conducted once during the exposure period for each test substance group (Groups 2–4). Samples were collected at approximately 1.8–1.9 L/min for 360, 150, and 60 min for the low, mid, and high concentration exposure groups, respectively. Following sample collection, substrates were re‐weighed, and the particle size was calculated based on the impactor stage cut‐offs. The particle‐size was expressed as the mass median aerodynamic diameter (MMAD) in microns and the geometric standard deviation (GSD).

The comet assay positive control, EMS, was dissolved in a 0.9% sodium chloride solution and administered once daily on study days 0 and 1 (days of necropsy) via oral gavage at a volume of 10 ml/kg body weight at a targeted concentration of 200 mg/kg body weight.

#### 2.2.3 Bronchoalveolar lavage

Six animals/dose in Groups 1 to 4 were sacrificed 1 or 18 h postexposure (note: in the range‐finding portion the animals were sacrificed immediately following exposure and 18 h postexposure). Animals treated with the positive control (Group 5) were necropsied 3 h after the second dose. Non‐fasted rats were anesthetized via inhalation of isoflurane and euthanized by exsanguination. BAL was performed in situ a total of 6 times with room temperature calcium‐ and magnesium‐free HBSS at a volume of 25 μl/g body weight up to 4 ml (per lavage). BALF from the first and second lavages was isolated in a refrigerated (4°C) centrifuge (2500 rpm) for 10 min. The supernatant fluid from the third through sixth lavages was decanted and discarded. The cell pellets obtained from the first and second lavages or third through sixth lavages were pooled separately and retained. Both cell pellets (from the first and second lavages; third‐sixth lavage) were resuspended in cold Roswell Park Memorial Institute (RPMI) medium with 10% fetal calf serum. All samples were placed on wet ice until samples were processed/analyzed.

#### 2.2.4 Cellular and molecular metrics

BALF from the first and second lavages was processed for clinical chemistry parameters (see below) and β‐glucuronidase quantification. BAL Clinical Chemistry (ALP, LDH, and total protein) was evaluated using a Siemens Advia 1800 Chemistry Analyzer (Siemens Medical Solutions USA, Malvern, PA).

Quantification of β‐glucuronidase by sandwich ELISA was conducted using the commercially available Rat GUSB/Beta Glucuronidase ELISA kit (LifeSpan BioSciences, Seattle, WA). Briefly, diluted BALF samples were added to 96‐well ELISA plates. Plates were sealed, agitated, and incubated for 90 min at 37°C. Detection antibody was added, and plates were incubated for an additional 60 min at 37°C. Residual liquid was removed, and plates were washed three times. Following washing, horseradish peroxidase (HRP) conjugate working solution was added and plates were incubated for 30 min at 37°C. Residual liquid was removed from the plate, washed, and 3,3',5,5'‐Tetramethylbenzidine (TMB) substrate was added. Plates were covered and incubated 15 min at 37°C protected from light followed by addition of a stopping solution and analyzed using SpectraMax M2 UV/Vis Florescence‐Chemiluminescence Microplate and Cuvette Reader (Molecular Devices, San Jose, CA) set at a wavelength of 450 nm.

Cell pellets were resuspended in 4.5 ml cold RPMI 1640 medium supplemented with 10% fetal calf serum. Cytology and other cellular metrics (see below) were conducted.

Total cell counts were obtained using a hemocytometer with cell viability assessed by trypan blue exclusion. Cytospin preparations of cell suspension were prepared and stained to evaluate differential cell counts. Differential cell counts of a minimum 200 nucleated cells/slide, when possible, were identified as macrophages, neutrophils, lymphocytes, eosinophils, or basophils.

A portion of the combined cell pellet from the first through sixth lavages was evaluated for Annexin V using a BD Biosciences, FITC Annexin V Apoptosis Detection Kit I and a flow cytometer. An aliquot of the resuspended cells from the BALF was added to DPBS (with Ca^2+^ and Mg^2+^) and was evaluated for Annexin V. The DPBS suspension was centrifuged (4°C, 500*g*, 10 min), cells were resuspended in binding buffer and centrifuged a second time. FITC‐Annexin V and PI (used to assess membrane integrity and hence cell viability) were added to the mixture and incubated while protected from light. When analyzed over time these measurements can track progression from early apoptosis (i.e., Annexin V positive, PI negative) to late apoptosis/necrosis (i.e., Annexin V positive, PI positive), which could suggest induction of apoptosis. Post‐incubation, the kit‐specific binding buffer was added, and sample acquisition was performed using a Cytomics FC 500 flow cytometer (Beckman Coulter, Brea, CA). Analysis was performed using FlowJo X Software (Version 10.0.8r1; Becton Dickinson, Ashland, OR).

#### 2.2.5 Comet assay in BAL, liver, and glandular stomach cells

Following excision and collection of tissues from necropsy, a section of the liver was placed in 3 ml of chilled mincing solution (HBSS, EDTA, and DMSO) and minced to release the cells. A section of the glandular stomach was placed in 1 ml of chilled mincing solution and scraped to release the cells. The liver and glandular stomach cell suspensions were strained through a cell strainer and stored on ice. The BALC were mixed with chilled mincing solution and stored on ice until processing. Cells from all preparations were processed for the comet assay as recommended by the 4th International Comet Assay Workshop (Hartmann et al., 2003), including alkaline processing and electrophoresis. Furthermore, experimental conduct was consistent with contemporary publications for the comet assay (Koppen et al., 2017; Moller et al., 2020).

Prepared slides (Trevigen 20‐well, Gaithersburg, MD; 0.5% low melt agarose) were stained with 1× SYBR Gold (Life Technologies, Carlsbad, CA). A minimum of three wells per tissue/animal/treatment were evaluated from the first five animals in each group. All slides were coded and scored blindly to eliminate potential scorer bias. Fifty randomly selected, nonoverlapping cells/slide/well were scored resulting in a total of 150 cells/tissue evaluated/animal for DNA damage. Comets were measured using image analysis software Comet IV (Perceptive Instruments, United Kingdom). As indicated in the OECD 489 test guideline, % tail DNA (also known as % tail intensity or % DNA in tail), was evaluated to assess DNA damage in this study. The data were processed as mean of median/well (*n* = 3/animal), resulting in a group mean for each treatment group. Slides were also examined for indication of heavily damaged cells (i.e., “clouds” or “hedgehogs”) and represented as a percentage of the total cells evaluated (at least 150/animal).

#### 2.2.6 Histopathology

Potential histopathologic alterations in liver and stomach were assessed from tissue whole mounts from control and MDI‐exposed rats. Following euthanasia sections of the liver (two lobes) and stomach were excised and fixed in 10% neutral buffered formalin. Tissues were processed into paraffin blocks, sectioned, mounted on glass microscope slides, and stained with hematoxylin and eosin (H & E). Stomach tissue was not scraped in a manner similar to the preparation of stomach cells for comet analysis prior to fixation/embedding. Stained histologic sections were examined by a veterinary pathologist via light microscopy. Severity grades were assigned to nonneoplastic histopathologic diagnoses in compliance with general guidelines (INHAND Program and SEND Initiative). Severity grades were assigned to a diagnosis to reflect a combination of considerations including the organ or tissue effect, the extent of the process (i.e., how many of its subordinate components are present), the distribution (i.e., focal to diffuse), and the degree of alteration in the examined histologic sections.

#### 2.2.7 Statistical analyses

Analyses were conducted using a two‐tailed test for minimum significance levels of 1% and 5%, comparing each MDI‐exposed group to the vehicle control group. BALF and BALC data were subjected to parametric one‐way ANOVA to determine intergroup differences. Statistically significant ANOVA (*p* ≤ .05) intergroup variance was further evaluated with a Dunnett's test to compare the MDI‐exposed groups to the vehicle control.

Comet assay analysis included parametric or nonparametric statistical methods based on variation between groups. Group variances for % tail DNA generated for the vehicle and MDI‐exposed groups was compared using Levene's test (*p* ≤ .05). Nonsignificant differences and variations between groups were assessed via a parametric one‐way ANOVA followed by a Dunnett's post hoc test (*p* ≤ .05). Linear regression analysis was used to determine a dose–response relationship (*p* ≤ .01). Pair‐wise comparison (Student's *t*‐test, *p* ≤ .05) was used to compare data from the positive control group versus vehicle control group.

## 3 RESULTS

### 3.1 Dose‐range finding study

#### 3.1.1 Chamber concentration and particle size

The concentration of aerosol present in each chamber was determined gravimetrically twice for the control, 10, and 20 mg/m^3^ exposure chambers and once for the 5 mg/m^3^ exposure chamber. The resulting TWA concentrations for the control, 5, 10, and 20 mg/m^3^ exposure chambers were determined to be 0, 4, 12, and 27 mg/m^3^. The actual chamber concentration values are presented in the text, tables, and figures.

Based on one determination each for the 4, 12, and 27 mg/m^3^ exposure chambers, the average MMAD values (± mean GSD) were 1.82 ± 1.71, 2.65 ± 2.20, and 2.48 ± 2.33 μm.

#### 3.1.2 General observations and body weight

All animals survived until scheduled necropsy (i.e., immediately following exposure or approximately 18 h postexposure). No clinical effects were noted during the exposure nor observations postexposure. Body weights for all exposure groups were similar to the control (data not shown).

### 3.2 Cellular and molecular metrics

#### 3.2.1 BAL fluid

**FIGURE 2:**
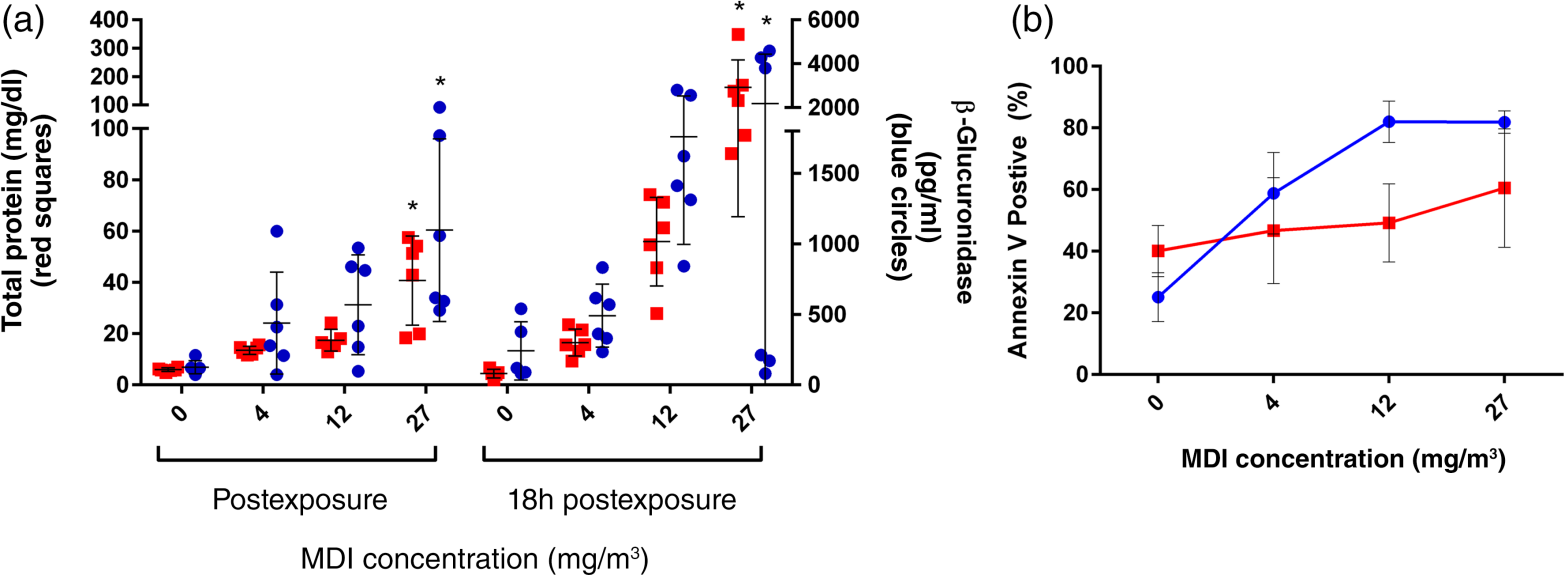
Dose‐range finding study. (a) Total protein (red squares) and β‐glucuronidase (blue circles) in BALF in samples taken immediately postexposure or 18 h after the end of exposure (*n* = 6/group, each dot represents an individual animal with bars representing mean and standard deviation/group). *p ≤ .05. (b) Apoptosis in BALC macrophages as measured by Annexin V staining in samples collected immediately postexposure (red squares) or 18 h after the end of exposure (blue circles). Data represent the mean and standard deviation for each group (*n* = 6/group) but were not statistically analyzed. BALC, bronchoalveolar lavage cells; BALF, bronchoalveolar lavage fluid

Concentration dependent increases in BALF total protein levels were observed in rats exposed to MDI (Figure 2a), which indicated alveolar‐capillary barrier damage and pulmonary toxicity. Compared to their respective controls, rats exposed to 4, 12, and 27 mg/m^3^ MDI had 2.3‐, 2.9‐, and 6.9‐fold increases immediately following exposure and higher magnitude increases, 3.8‐, 12.7‐, and 36.8‐fold, respectively 18 h after exposure. Though a statistical significance was observed in LDH level at 4 and 27 mg/m^3^ 18 h after exposure, the biological significance was not clear given the magnitude of change and lack of dose–response (Table 1). No treatment related alterations in ALP levels were identified either immediately following exposure or 18 h postexposure (Table 1).

**TABLE 1:** Dose‐range finding study: BALF and BALC measures

MDI (mg/m^3^)	*N*	LDH (U/L)	ALP (U/L)	Total cells (×10^6^/ml)
*Postexposure*				
0	6	30 ± 2	25 ± 5	1.374 ± 0.563
4	6	28 ± 4	31 ± 8	0.814 ± 0.535
12	6	37 ± 5	25 ± 6	0.648 ± 0.265
27	6	39 ± 8	29 ± 7	0.734 ± 0.550
*18‐h postexposure*				
0	6	22 ± 5	16 ± 13	0.416 ± 0.129
4	6	37 ± 5^a^	15 ± 10	1.467 ± 0.384^a^
12	6	28 ± 5	21 ± 13	0.404 ± 0.212
27	6	47 ± 14^a^	20 ± 4	1.244 ± 0.274

*Note*: Mean ± standard deviation.

Abbreviations: ALP, alkaline phosphatase; BALC, bronchoalveolar lavage cells; BALF, bronchoalveolar lavage fluid; LDH, lactate dehydrogenase; MDI, 1,1′‐methylenebis(4‐isocyanatobenzene).

a Dunnett's test statistically identified at *p* ≤ .05.

Concentration dependent increases were also observed in β‐glucuronidase levels in rats exposed to MDI (Figure 2a), which was indicative of macrophage activation. Compared to their respective controls, rats exposed to 4, 12, and 27 mg/m^3^ MDI had 3.5‐, 4.6‐, and 8.8‐fold increases immediately following exposure and the effect persisted to 18 h after exposure, with 2.4‐, 8.5‐, and 10.5‐fold increases, respectively.

Statistical significances were identified in GSH, GSSG, and GSH/GSSG ratio in rats exposed to MDI (Supplemental Table 1); however, the biological significance was not clear given the magnitude of change and lack of dose–response.

#### 3.2.2 BAL cells

No meaningful changes in total white blood cell count were observed either immediately following exposure or 18 h after exposure. Cytospins were evaluated for % cellular composition including pulmonary alveolar macrophages (PAM), polymorphonuclear neutrophils (PMN), lymphocytes (LYM), and eosinophils (EOS). Concentration dependent increases in %PMN were observed in rats exposed to MDI (Supplemental Table 2) which was indicative of neutrophilic inflammation at the point‐of‐contact of MDI exposure. Compared to their respective controls, rats exposed to 4, 12, and 27 mg/m^3^ MDI had 0.9‐, 2.0‐, and 4.1‐fold increase in %PMN immediately following exposure and the effect persisted to 18 h after exposure, with 0.7‐, 3.6‐, and 4.4‐fold increases, respectively.

Analysis of gene expression data showed concentration‐dependent responses of a number of cytokine‐related markers, including *Mcp‐1*, *Il‐6*, *Il‐1a*, and *Il‐10* in the 18 h postexposure groups (Supplemental Table 3) but not selected genes associated with apoptosis or oxidative stress.

The Annexin V assay was conducted to detect the percentage of apoptotic BALC following inhalation exposure of MDI. Immediately after exposure, the average percent early apoptotic cells (Annexin (+)/PI (−) cells) in total nucleated BALC was increased 1.1‐, 1.2‐, and 1.4‐fold at 4, 12, and 27 mg/m^3^, respectively, compared to 40.8% in the control group. This cytotoxic effect progressed to a higher magnitude at 18 h postexposure. The average percent early apoptotic cells was increased 1.9‐, 2.4‐, and 2.8‐fold at 4, 12, and 27 mg/m^3^, respectively, compared to 22.4% in the control group. Individual macrophage and neutrophil were analyzed separately (Figure 2b and Supplemental Table 4).

Under the conditions of this experiment, Caspase‐3 (+) cells were low and were not captured as a separate cluster from the Caspase‐3 (−) cells in the flow cytometric analysis (data not shown).

In total, based on the above cellular and molecular markers, a concentration of 4 mg/m^3^ was considered the maximum noninflammatory concentration, hence target concentrations of 2, 5, and 11 mg/m^3^ were selected for the main study, which included the comet end point.

### 3.3 Main study

#### 3.3.1 Chamber concentration and particle size

Calculated exposure concentrations were 2.5 ± 0.05, 4.9 ± 0.38, and 12.0 ± 0.4 mg/m^3^ (study mean ± standard deviation) for exposure groups 2, 3, and 4, respectively. Measured aerosol particle sizes for groups 2, 3, and 4 were 3.7 ± 2.23, 1.8 ± 2.86, and 2.7 ± 2.20 μm (MMAD ± GSD). The similar and overlapping aerosol particle sizes between treatment groups and phases of the study indicate similar lung, inhalation, and exposure parameters.

#### 3.3.2 General observations and body weight

All animals survived to the scheduled necropsy. There were no test substance‐related clinical observations or body weight changes noted in any groups.

### 3.4 Cellular and molecular metrics

#### 3.4.1 BAL fluid

**FIGURE 3:**
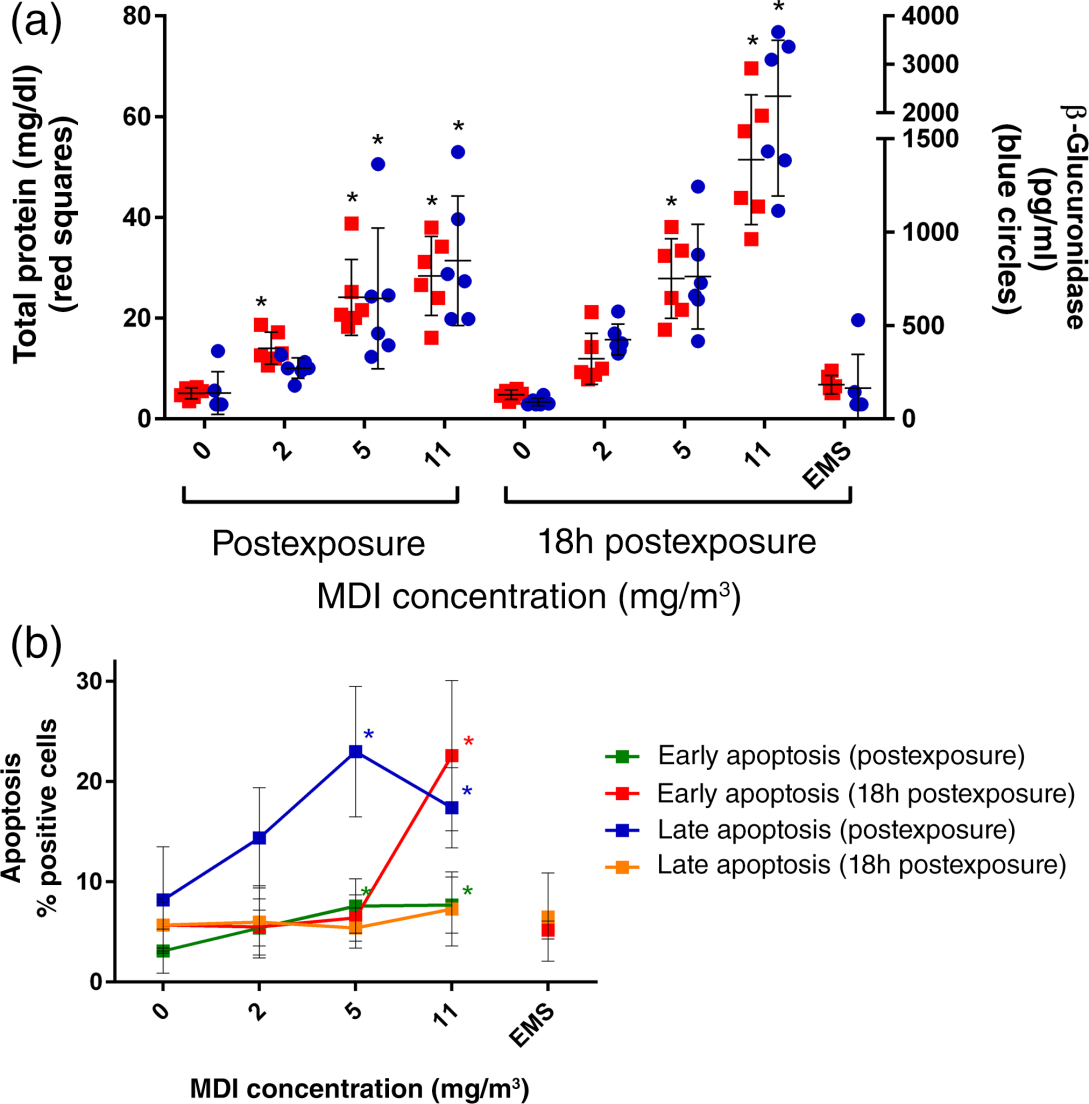
Main study. (a) Total protein (red squares) and β‐glucuronidase (blue circles) in BALF in samples taken postexposure or 18 h after the end of exposure (*n* = 6/group, each dot represents an individual animal with bars representing mean and standard deviation/group). (b) Annexin V assessment for early‐ or late‐stage apoptosis in total BALC in samples collected postexposure or 18 h after the end of exposure. Data represent the mean and standard deviation for each group (*n* = 6/group). *p ≤ .05. BALC, bronchoalveolar lavage cells; BALF, bronchoalveolar lavage fluid

MDI‐associated differences in BALF clinical chemistry parameters were observed 1‐h postexposure (Figure 3a). Dose‐dependent increases in total protein were observed in MDI‐exposed animals, with 2.7‐, 4.7, and 5.6‐fold increase at targeted concentrations of 2, 5, and 11 mg/m^3^, respectively. Dose‐dependent increases were also observed in β‐glucuronidase concentrations, with 2.0‐, 4.7, and 6.1‐fold increase at 2, 5, and 11 mg/m^3^, respectively.

As observed in the dose range finding study, the impact on BALF total protein and β‐glucuronidase persisted to 18 h postexposure and with a larger induction versus the shorter time point (Figure 3a). Compared to their respective controls, rats exposed to targeted concentrations of 2, 5, and 11 mg/m^3^ MDI had 2.5‐, 5.8‐, and 10.7‐fold increase in total protein, and 4.7‐, 8.4‐, and 25.8‐fold increase in β‐glucuronidase concentrations.

Statistical significances were identified in LDH and ALP in rats exposed to MDI (Table 2); however, the biological significance was not clear given the magnitude of change or lack of dose–response.

**TABLE 2:** Main study: BALF and BALC measures

MDI (mg/m^3^)	*N*	LDH (U/L)	ALP (U/L)	Total cells (×10^6^/ml)
*Postexposure*				
0	6	44 ± 10.3	52 ± 7.7	5.78 ± 3.70
2	6	59 ± 1135	68 ± 15.4	2.89 ± 0.87
5	6	72 ± 19.2^a^	68 ± 10.9	3.09 ± 1.05
11	6	69 ± 18.2^a^	80 ± 24.6^a^	4.82 ± 2.13
*18‐h postexposure*				
0	6	46 ± 9.9	74 ± 14.5	3.59 ± 1.64
2	6	44 ± 12.9	54 ± 12.6^a^	4.07 ± 2.19
5	6	53 ± 7.1	78 ± 17.0	3.78 ± 2.03
11	6	61 ± 19.3	80 ± 9.2	3.22 ± 1.45
EMS (200 mkd)	6	39 ± 7.1	52 ± 8.6^a^	3.57 ± 1.14

*Note*: Mean ± standard deviation.

Abbreviations: ALP, alkaline phosphatase; BALC, bronchoalveolar lavage cells; BALF, bronchoalveolar lavage fluid; EMS, ethyl methanesulfonate; LDH, lactate dehydrogenase; MDI, 1,1′‐methylenebis(4‐isocyanatobenzene).

a Dunnett's test statistically identified at *p* ≤ .05.

#### 3.4.2 BAL cells

Alterations in BAL cytology were also in the same pattern as the dose range finding study. No meaningful changes were observed in total white blood cell count either 1‐h or 18 h postexposure. Concentration dependent increases in %PMN were observed in rats exposed to MDI (Supplemental Table 5). Compared to their respective controls, rats exposed to targeted concentrations of 2, 5, and 11 mg/m^3^ MDI had 2.2‐, 2.3‐, and 5.7‐fold increases in %PMN 1‐h postexposure and the effect persisted to 18 h after exposure at higher magnitude, with 12.8‐, 6.9‐, and 24.5‐fold increases, respectively.

Apoptosis was also observed in the same pattern as the dose range finding study. At 1‐h postexposure, the average percent early apoptotic cells was increased 1.7‐, 2.5‐, and 2.5‐fold at 2, 5, and 11 mg/m^3^, respectively, compared to 3.1% in the control group (Figure 3b). This cytotoxic effect progressed to a higher magnitude at 11 mg/m^3^ at 18 h postexposure. The average percent early apoptotic cells was increased 1.0‐, 1.1‐, and 4.0‐fold at 2, 5, and 11 mg/m^3^, respectively, compared to 5.7% in the control group.

#### 3.4.3 Comet assay

**FIGURE 4:**
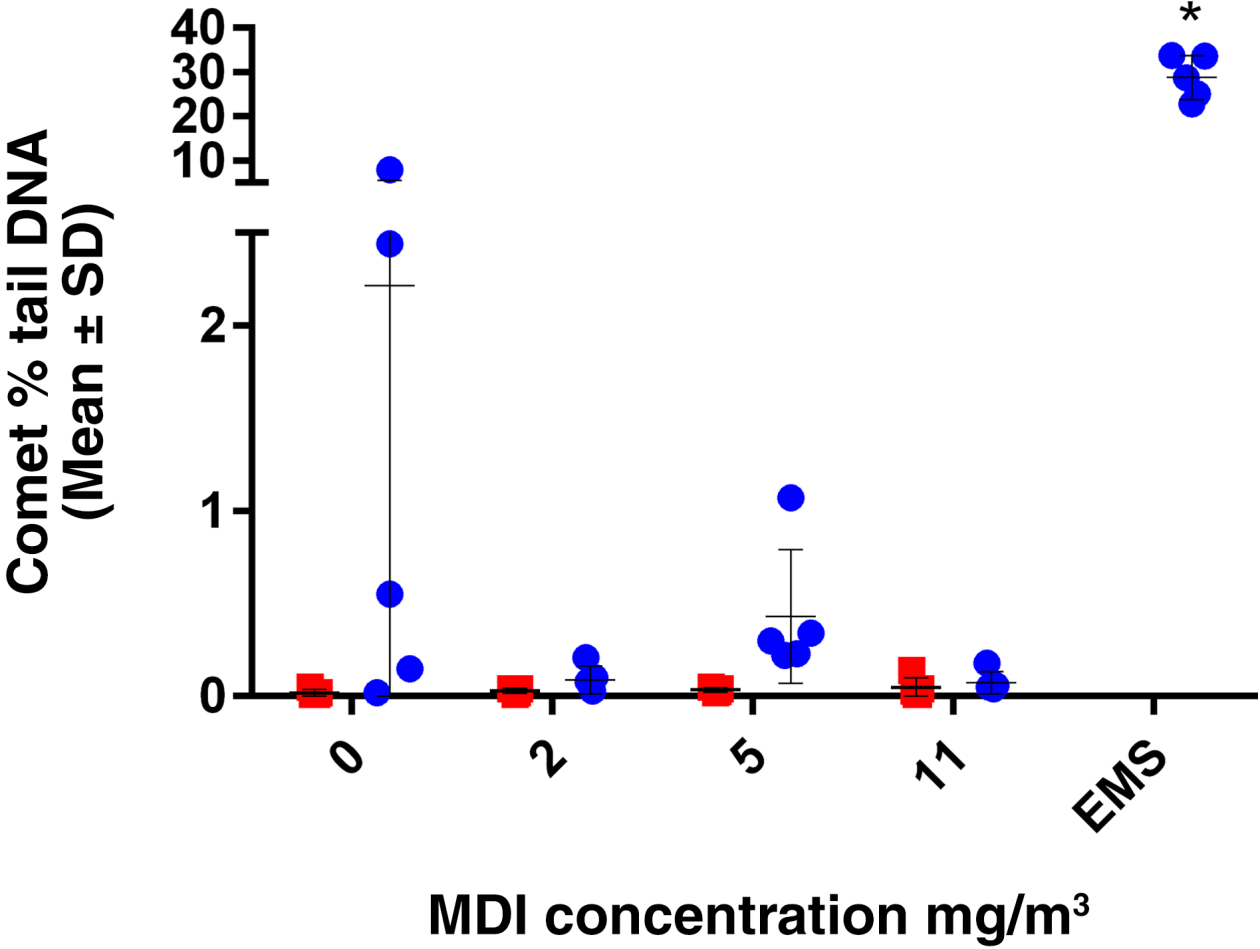
Comet assay. BALC were collected postexposure (red squares) or 18 h after the end of exposure (blue circles, *n* = 5/group, each dot represents an individual animal with bars representing mean and standard deviation/group). Liver and glandular stomach comet data are presented in Table 3. Positive control tissue was collected 3 h after the second oral administration of EMS. *p ≤ .05. BALC, bronchoalveolar lavage cells; EMS, ethyl methanesulfonate

The comet assay was conducted on BALC, glandular stomach, and liver from rats exposed to either filtered air (0 mg/m^3^) or aerosolized MDI at both 1 and 18 h postexposure. The mean number of clouds for each tissue in the filtered air control groups (1 and 18 h postexposure) were less than the 30% threshold, although one animal from the 18 h postexposure in the liver exceeded the 30% threshold; this animal/tissue was replaced with another in that same treatment group. Exposure to MDI did not induce a statistically significant increase in DNA damage as represented by % tail DNA in BALC, glandular stomach, or liver cells at any concentrations or postexposure time‐points (Table 3 and Figure 4). The positive control, EMS (200 mg/kg body weight), induced a significant increase in % tail DNA compared to the filtered air control group in all tissues examined. Therefore, under the conditions of this study, the administration of MDI at targeted concentrations of 2, 5, and 11 mg/m^3^ did not induce a significant increase in DNA damage in BALC, liver, or glandular stomach cells relative to the concurrent filtered air control at 1 or 18 h post exposure.

**TABLE 3:** Main study: Comet assay tail DNA (%)

MDI (mg/m^3^)	*N*	Liver	Glandular stomach
*Postexposure*			
0	5	0.15 ± 0.09	10.35 ± 4.39
2	5	0.19 ± 0.13	4.75 ± 3.45
5	5	0.27 ± 0.37	5.27 ± 4.29
11	5	0.22 ± 0.13	8.91 ± 3.77
*18‐h postexposure*			
0	5	0.05 ± 0.03	20.18 ± 5.04
2	5	0.08 ± 0.08	18.70 ± 5.55
5	5	0.03 ± 0.02	16.22 ± 8.71
11	5	0.16 ± 0.14	20.42 ± 12.77
EMS (200 mkd)	5	37.82 ± 4.61^a^	51.35 ± 4.72^a^

*Note*: Mean ± standard deviation.

Abbreviations: EMS, ethyl methanesulfonate; MDI, 1,1′‐methylenebis(4‐isocyanatobenzene).

a Dunnett's test statistically identified at *p* ≤ .05.

#### 3.4.4 Histopathology

Stained (H & E) sections of liver and glandular stomach were assessed for test material‐related histopathologic alterations via light microscopy. MDI‐related microscopic findings were noted in the liver of the 2, 5, and 11 mg/m^3^ groups at 1 h postexposure and included “minimal to mild” increased mitotic figures in hepatocytes throughout the parenchyma (Supplemental Table 6). Similar findings were not observed 18 h postexposure, although increased mitoses were observed in the EMS (positive control group). No other test material‐related histopathologic changes were observed in the liver or glandular stomach.

## 4 DISCUSSION

Cytotoxicity assessment is essential for proper conduct of an *in vivo* comet assay. As indicated in OECD_TG489 (2016) and supporting documents, the most widely used indication of cellular/tissue homeostasis is histopathology. While there remains a general need for standardized (and ideally an objective and quantitative) histopathological evaluation format (Burlinson et al., 2007; Speit et al., 2015) it has long‐been appreciated that histopathological manifestations of necrosis/cytotoxicity do not readily align with the acute dosing and tissue‐collection paradigm of an *in vivo* comet assay. Specifically, histopathological evidence of cell/tissue death is not apparent for 12–24 h (Cobb et al., 1996; Fink & Cookson, 2005; Majno & Joris, 1995) and hence would require a separate group of animals for evaluation. Furthermore, not all cell types utilized in the comet assay are amenable to histopathology, cellular, and/or tissue‐contextual evaluation, for example, when utilizing nucleated peripheral blood or lung lavage cells. In these cases, and perhaps all comet evaluations, other measures of cellular status such as apoptosis, necrosis, and general metabolic competency should be considered.

Although the potential confounding effects of apoptosis/necrosis on interpretation of the comet assay have been discussed, conclusions from the 5th International Workshop on Genotoxicity Testing (Rothfuss et al., 2011) indicated no clear data to support (or refute) the inclusion of appropriate, specific cytotoxicity measures for improved interpretation of the *in vivo* comet assay. While the measures investigated in that publication were largely associated with comet conduct (e.g., hedgehogs, neutral diffusion) or tissue function (e.g., liver enzymes in blood), the use of cellular measures (e.g., apoptosis or viability) was not specifically investigated. Lastly, the authors were not able to identify data that indicate cytotoxicity, by itself, that increase or decrease DNA migration (i.e., comet response).

MDI is a highly reactive chemical which results in pulmonary inflammation after acute inhalation exposure and increases the incidence of lung tumors in rats at 6 mg/m^3^ (but not lower) after chronic exposure, and the weight of evidence supports a non‐genotoxic mode of action for the carcinogenicity (Pauluhn et al., 2001). In an unpublished contemporary comet study described in the EU REACH dossier (ECHA, 2016), male Wistar rats were exposed via inhalation for 3 or 6 h at concentrations of 10, 20, 100, or 180 mg/m^3^ MDI and BALC were collected and analyzed. In addition, a comet positive control group was administered 50 mg/kg body weight methyl nitrosourea (MNU) via oral gavage, and an inhalation particulate comparison group was administered paraffin solid aerosol (a non‐genotoxic reference compound) via inhalation at 25 mg/m^3^ for 6 h. That study was conducted prior to the finalization of the OECD 489 TG, but conduct was largely consistent with the guideline recommendations. Similar to the results presented herein, MDI caused a dose‐responsive increase in LDH and protein concentration in BALF, resulted in increased inflammation (% PMN and foamy macrophages), and increased apoptosis. Interestingly, paraffin also increased Annexin V positive cells and inflammation. With respect to the comet assay, at MDI concentrations ≥ 20 mg/m^3^ and paraffin (25 mg/m^3^) both substances caused an increase in % tail DNA, suggesting the positive comet response in that study was secondary to non‐genotoxic properties of a given chemical or particulate.

In this study, we sought to further assess the *in vivo* genotoxic potential of MDI via inhalation exposure and investigated potential cellular and molecular changes up to and including inflammation and apoptosis to aid in dose selection for appropriate evaluation in the *in vivo* comet assay. In the conduct of this two‐phase, GLP‐ and OECD guideline‐compliant work substantial cellular and molecular alterations were noted in response to MDI administration. Specifically, in the dose range finding study, a single 6h exposure to MDI resulted in concentration‐ and time‐dependent increases in alveolar‐capillary barrier damage, inflammation, and apoptosis; the 12 and 27 mg/m^3^ exposures evaluated 18 h postexposure indicated the most robust responses. In both phases of the work described here two time points were utilized to characterize the potential differences in cellular response for point‐of‐contact and systemic tissues. Based on the magnitude of the responses for the measured endpoints 4 mg/m^3^ represented the maximum noninflammatory concentration (and hence by extension was used to guide concentration‐selection and define an MTD) of respirable MDI aerosol in male Wistar rats exposed for 6 h. Concentrations ≥ 20 mg/m^3^ resulted in excessive respiratory tract irritation and toxicity. Given these results, targeted concentrations of 2, 5, and 11 mg/m^3^ were selected for the main *in vivo* comet study.

In acute and chronic inhalation studies, characterization of lung status and/or injury is routinely assessed by both histological analysis and biochemical/cytological content of BALF. Among the most common measures, total protein in BALF is used to assess the integrity of the alveolar‐capillary barrier (Henderson et al., 1987; OECD, 2018). In fact, a number of prior publications on related polyisocyanate compounds have shown BALF total protein to be the most sensitive measure of pulmonary toxicity (Ma‐Hock et al., 2007; Pauluhn, 2004). In both the range‐finding and main studies for MDI, we saw substantial, dose‐responsive, and reproducible increases in total protein content of the BALF, with greater than a 10‐fold increase at the 12 or 11 mg/m^3^ concentration in the samples collected 18 h after cessation of exposure. An additional measure of alveolar homeostasis utilized in this study was BALF β‐glucuronidase, which is indicative of macrophage status and is increased as a result of phagocytosis and/or cytotoxicity to phagocytotic cells (Henderson et al., 1987; Unanue, 1976). In the range‐finding portion, this value was increased ~5‐fold and 8.5‐fold at 12 mg/m^3^ immediately after treatment and 18 h postexposure, respectively, and increased to ~10‐fold at the 27 mg/m^3^ concentration at those time points. Similar, and in many cases greater, induction was noted for the β‐glucuronidase end point in the main study after MDI administration.

In both the dose range finding study and main study Annexin‐V staining was utilized and generally indicated a dose‐responsive increase in the number of apoptotic cells. Specifically in the dose range finding study, characterization of the macrophage and neutrophil population was analyzed separately for early‐stage apoptosis with the macrophages showing an ~1.5‐fold increase in the 27 mg/m^3^ samples collected immediately after exposure and an ~3‐fold increase in the 12 and 27 mg/m^3^ samples collected 18 h after completion of exposure (Supplemental Table 4 and Figure 2b). Annexin V staining in the neutrophils decreased with increasing MDI concentration, presumably as a result of neutrophil influx in response to tissue damage during exposure. In the main study Annexin V staining largely mirrored the response in the range‐finding phase, with a ~4‐fold increase in 11 mg/m^3^ samples collected 18 h posttreatment, although no separate determination for macrophage or polymorphonuclear cells was made in this phase of the study. Interestingly, however, in the range‐finding/method development stage not all apoptosis metrics were consistent or demonstrated a clear response. For example, when compared to the Annexin V response, Caspase‐3 (data not shown) or the gene expression biomarkers (*Bcl‐2* or *Bax*, Supplemental Table 3) were less definitive. Previous work has reported an apoptosis/necrosis signature correlated with increases in a comet tail and suggested not to be readily discerned from a true genotoxicity signature (Guerard et al., 2014; Lorenzo et al., 2013; OECD, 2014). Thus, dose levels inducing substantial apoptosis should be avoided in an *in vivo* comet assay, otherwise the genotoxicity evaluation might be confounded by apoptosis. In this study, an inflammatory and apoptotic signature was present in BALF after MDI administration at concentrations tested for comet responses. Traditional animal‐ or histopathological‐based systemic toxicity (i.e., MTD) measures were not present; however, point‐of‐contact tissues were robustly altered.

Due to the chemical nature of MDI and the aerosol generating apparatus used in this study, an interesting comparison can be made on the biological response to administration of particulates, specifically using diesel exhaust and carbon black (Kyjovska et al., 2015). In that study no increase in the comet response (as % tail DNA) was noted at any dose level in BALC, although carbon black did increase the protein content in the BALF. Although the authors noted some increases in metrics (e.g., comet tail length) and time points (e.g., 3 and 28 days posttreatment) that diverge from the recommendations of OECD 489 TG and typical study conduct, overall, the results suggest that altered alveoli integrity does not necessarily equate to DNA damage. These results for diesel particulate are consistent with the data presented herein where a number of molecular and cellular metrics indicated substantial alterations as a result of MDI inhalation exposure, yet no appreciable increase in comet in BALC was noted and contrast to the response to particulate paraffin described earlier (ECHA, 2016).

In conclusion, specific cellular and molecular metrics may provide a relevant and timely measure of cell health. In cases where histopathological examination may not be informative or feasible, supplemental clinical measures can be used to characterize cytotoxicity. Under the conditions of this study, MDI inhalation did not induce genotoxicity (i.e., DNA strand breaks) at the point of contact in lung (BALC) or distally in liver or glandular stomach at concentrations inducing inflammatory responses and apoptosis in the lung.

## AUTHOR CONTRIBUTIONS

Zhiying Ji, Matthew W. Koehler, and Matthew J. LeBaron contributed to execution and interpretation of the range‐finding experiments. All authors contributed to manuscript preparation and approved the final article.

## Supporting information


**Supplemental Table 1**. Dose‐range finding study: GSH/GSSG measures
**Supplemental Table 2**. Dose‐range finding study: Total BAL cell differential
**Supplemental Table 3**. Dose‐range finding study: Gene expression
**Supplemental Table 4**. Dose‐range finding study: Apoptosis in BAL macrophages and neutrophils
**Supplemental Table 5**. Main study: Total BAL cell differential
**Supplemental Table 6**. Main study: Histopathologic evaluation of liver and stomach
