# Supplementary material for: Assessment of cellular and molecular metrics for dose selection in an in vivo comet assay: A case study with MDI
Source: Environ Mol Mutagen. 2021 Aug 26;62(8):446–57. doi: 10.1002/em.22457 (PMC9290646; doi:10.1002/em.22457)
Supplement: Supplementary file 1 — Supplemental Table 1. Dose‐range finding study: GSH/GSSG measures Supplemental Table 2. Dose‐range finding study: Total BAL cell differential Supplemental Table 3. Dose‐range finding study: Gene expression Supplemental Table 4. Dose‐range finding study: Apoptosis in BAL macrophages and neutrophils Supplemental Table 5. Main study: Total BAL cell differential Supplemental Table 6. Main study: Histopathologic evaluation of liver and stomach [file EM-62-446-s001.docx]

**Supplemental Information**

**Supplemental Table 1. Dose-Range Finding Study: GSH/GSSG Measures**

| **MDI  (mg/m^3^)** | **N** | **GSH**  **(nmol)** | **GSSG**  **(nmol)** | **GSH/GSSG** |
| --- | --- | --- | --- | --- |
| **Post Exposure** | | | | |
| 0 | 6 | 6.02 ± 1.88 | 0.86 ± 0.24 | 7.08 ± 1.43 |
| 4 | 6 | 5.19 ± 2.80 | 1.04 ± 0.33 | 4.77 ± 1.23* |
| 12 | 6 | 7.98 ± 2.46 | 1.54 ± 0.67 | 5.86 ± 2.33* |
| 27 | 6 | 10.95 ± 9.29 | 3.32 ± 3.20 | 3.53 ± 0.81* |
| **18-Hours Post Exposure** | | | | |
| 0 | 6 | 3.42 ± 1.67 | 0.54 ± 0.21 | 6.27 ± 2.18 |
| 4 | 6 | 7.45 ± 2.10* | 0.87 ± 0.08 | 8.80 ± 1.04 |
| 12 | 6 | 4.28 ± 0.50 | 1.11 ± 0.43* | 4.27 ± 1.38 |
| 27 | 6 | 6.96 ± 2.32* | 2.94 ± 0.77* | 2.49 ± 1.04* |

Mean ± SD

*Dunnett’s test statistically identified at p < 0.05

**Supplemental Table 2. Dose-Range Finding Study: Total BAL Cell Differential**

| **MDI  (mg/m^3^)** | **N** | **%PAM** | **%PMN** | **%LYM** | **%EOS** |
| --- | --- | --- | --- | --- | --- |
| **Post Exposure** | | | | | |
| 0 | 6 | 99.4 ± 0.6 | 1.0 ± 0.7 | 0.8 ± 0.4 | 0.0 ± 0.0 |
| 4 | 6 | 99.2 ± 0.9 | 0.9 ± 1.0 | 0.0 ± 0.0 | 0.5 ± 0.0 |
| 12 | 6 | 97.3 ± 1.5 | 2.0 ± 1.5 | 1.1 ± 0.9 | 0.0 ± 0.0 |
| 27 | 6 | 95.5 ± 4.1* | 4.1 ± 3.6* | 1.3 ± 0.4 | 0.0 ± 0.0 |
| **18-Hours Post Exposure** | | | | | |
| 0 | 6 | 98.1 ± 1.7 | 2.3 ± 1.6 | 0.0 ± 0.0 | 0.0 ± 0.0 |
| 4 | 6 | 98.6 ± 1.1 | 1.5 ± 1.1 | 0.5 ± 0.0 | 0.5 ± 0.0 |
| 12 | 6 | 91.6 ± 6.3* | 8.2 ± 5.9* | 0.0 ± 0.0 | 0.8 ± 0.0 |
| 27 | 6 | 89.8 ± 4.3* | 10.2 ± 4.3* | 0.5 ± 0.0 | 0.0 ± 0.0 |

Mean ± SD

*Dunnett’s test statistically identified at p < 0.05

**Supplemental Table 3. Dose-Range Finding Study: Gene Expression**

| **MDI  (mg/m^3^)** | **N** | ***Nrf2a*** | ***Bax*** | | ***Ifn-γ*** | ***Bcl-2*** | ***Aox1*** | ***Mcp-1*** | ***Gpx1*** | ***Tnf-α*** | ***Il-6*** | ***Il-1a*** | | ***Il-10*** | | ***Mip-2*** |
| --- | --- | --- | --- | --- | --- | --- | --- | --- | --- | --- | --- | --- | --- | --- | --- | --- |
| **Post Exposure** | | | | | | | | | | | | | | | | |
| 0 | 6 | 1 | 1 | NQ | | 1 | 1 | 1 | 1 | 1 | 1 | 1 | 1 | | 1 | |
| 4 | 6 | 0.8 | 0.8 | NQ | | 1 | 0.7 | 1.5 | 0.7 | 1 | 0.4 | 0.8 | 0.5 | | 1.3 | |
| 12 | 6 | 0.7 | 0.7 | NQ | | 1.2 | 3.3 | 2.7 | 0.5 | 0.9 | 1.2 | 0.8 | 1.1 | | 2.9 | |
| 27 | 6 | 0.7 | 0.6 | NQ | | 1.1 | 1.4 | 2.1 | 0.5 | 0.9 | 1.1 | 0.5 | 1.4 | | 2.4 | |
| **18-Hours Post Exposure** | | | | | | | | | | | | | | | | |
| 0 | 6 | 1 | 1 | NQ | | 1 | 1 | 1 | 1 | 1 | 1 | 1 | 1 | | 1 | |
| 4 | 6 | 1.2 | 1 | NQ | | 0.9 | 0.9 | 1.3 | 0.9 | 1 | 3 | 0.9 | 0.9 | | 0.6 | |
| 12 | 6 | 1.1 | 1.2 | NQ | | 0.9 | 0.5 | 7.3 | 0.8 | 1.1 | 2.5 | 1.4 | 1.5 | | 1 | |
| 27 | 6 | 1.1 | 1.8 | NQ | | 1 | 0.7 | 63.8 | 0.7 | 1.4 | 8.1 | 2.7 | 5.7 | | 2.4 | |

Fold Change relative to respective control; not statistically analyzed

NQ = Not Quantifiable

**Supplemental Table 4. Dose-Range Finding Study: Apoptosis in BAL Macrophages and Neutrophils**

|  |  | **Macrophages** | |  | **Neutrophils** | |
| --- | --- | --- | --- | --- | --- | --- |
| **MDI  (mg/m^3^)** | **N** | **No. of Cells  Analyzed** | **% Annexin V (+) /PI (-)** |  | **No. of Cells  Analyzed** | **% Annexin V (+) /PI (-)** |
| **Post Exposure** | | | | | | |
| 0 | 6 | 1951 ± 257 | 40.1 ± 8.3 |  | 56 ± 25 | 65.3 ± 6.0 |
| 4 | 6 | 1472 ± 503 | 46.7 ± 17.2 |  | 69 ± 21 | 46.3 ± 8.4 |
| 12 | 6 | 1971 ± 628 | 49.2 ± 12.7 |  | 108 ± 24 | 30.0 ± 9.6 |
| 27 | 6 | 2150 ± 386 | 60.5 ± 19.2 |  | 107 ± 35 | 27.2 ± 9.4 |
| **18-Hours Post Exposure** | | | | | | |
| 0 | 6 | 3829 ± 1193 | 25.1 ± 7.9 |  | 1421 ± 1878 | 15.7 ± 7.7 |
| 4 | 6 | 4159 ± 1591 | 58.8 ± 13.2 |  | 1889 ± 1240 | 10.0 ± 0.8 |
| 12 | 6 | 20398 ± 12560 | 82.0 ± 6.7 |  | 11197 ± 3450 | 9.4 ± 1.3 |
| 27 | 6 | 13028 ± 3253 | 81.9 ± 3.6 |  | 5407 ± 3172 | 12.8 ± 2.9 |

Mean ± SD; not statistically analyzed

**Supplemental Table 5. Main Study: Total BAL Cell Differential**

| **MDI  (mg/m^3^)** | **N** | **%PAM** | **%PMN** | **%LYM** | **%EOS** |
| --- | --- | --- | --- | --- | --- |
| **Post Exposure** | | | | | |
| 0 | 6 | 98.0 ± 1.5 | 0.58 ± 0.59 | 1.08 ± 1.02 | 0.33 ± 0.52 |
| 2 | 6 | 97.5 ± 1.9 | 1.25 ± 1.08 | 0.25 ± 0.42 | 0.00 ± 0.00 |
| 5 | 6 | 98.3 ± 0.7 | 1.33 ± 0.41 | 0.42 ± 0.80 | 0.00 ± 0.00 |
| 11 | 6 | 96.0 ± 2.6 | 3.33 ± 2.81* | 0.42 ± 0.49 | 0.25 ± 0.61 |
| **18-Hours Post Exposure** | | | | | |
| 0 | 6 | 96.3 ± 3.1 | 0.17 ± 0.41 | 3.42 ± 3.09 | 0.08 ± 0.20 |
| 2 | 6 | 95.9 ± 3.0 | 2.17 ± 2.56 | 1.83 ± 1.21 | 0.08 ± 0.20 |
| 5 | 6 | 97.9 ± 1.2 | 1.17 ± 0.75 | 0.75 ± 0.42 | 0.17 ± 0.41 |
| 11 | 6 | 94.1 ± 3.3 | 4.17 ± 3.11* | 1.58 ± 1.07 | 0.17 ± 0.29 |

Mean + SD

*Dunnett’s test statistically identified at p < 0.05

**Supplemental Table 6. Main Study: Histopathologic Evaluation of Liver and Stomach**

|  | **Post Exposure** | | | | **18h Post Exposure** | | | | |
| --- | --- | --- | --- | --- | --- | --- | --- | --- | --- |
|  | **MDI (mg/m^3^)** | | | | **MDI (mg/m^3^)** | | | | |
|  | **0** | **2** | **5** | **11** | **0** | **2** | **5** | **11** | **EMS** |
| Liver | | | | | | | | | |
| N^a^ | 6 | 6 | 6 | 6 | 6 | 6 | 6 | 6 | 6 |
| Increased mitoses | 0 | 2 | 2 | 4 | 0 | 0 | 1 | 0 | 6 |
| Minimal | - | 2 | 1 | 3 | - | - | 1 | - | 3 |
| Mild | - | 0 | 1 | 1 | - | - | 0 | - | 2 |
| Moderate | - | 0 | 0 | 0 | - | - | 0 | - | 1 |
| Stomach, glandular | | | | | | | | | |
| N^a^ | 6 | 6 | 6 | 6 | 6 | 6 | 6 | 6 | 6 |
| Unremarkable | 6 | 6 | 6 | 6 | 6 | 6 | 6 | 6 | 6 |
| Stomach, non-glandular | | | | | | | | | |
| N^a^ | 6 | 6 | 6 | 6 | 6 | 6 | 6 | 6 | 6 |
| Unremarkable | 6 | 6 | 6 | 6 | 5 | 6 | 6 | 6 | 4 |
| Neutrophil Infiltrate Minimal | 0 | 0 | 0 | 0 | 1 | 0 | 0 | 0 | 2 |

^a^Number of tissues examined from each group
